# The Impact of Environment on Gait Assessment: Considerations from Real-World Gait Analysis in Dementia Subtypes

**DOI:** 10.3390/s21030813

**Published:** 2021-01-26

**Authors:** Ríona Mc Ardle, Silvia Del Din, Paul Donaghy, Brook Galna, Alan J Thomas, Lynn Rochester

**Affiliations:** 1 Translational and Clinical Research Institute, Faculty of Medical Sciences, Newcastle University, Newcastle Upon Tyne NE4 5PL, UK; silvia.del-din@ncl.ac.uk (S.D.D.); paul.donaghy@ncl.ac.uk (P.D.); brook.galna@ncl.ac.uk (B.G.); alan.thomas@ncl.ac.uk (A.J.T.); lynn.rochester@ncl.ac.uk (L.R.); 2 School of Biomedical, Nutritional and Sport Sciences, Newcastle University, Newcastle Upon Tyne NE1 7RU, UK; 3 Newcastle Upon Tyne Hospital NHS Foundation Trust, Newcastle Upon Tyne NE7 7DN, UK

**Keywords:** wearable technology, gait, dementia, accelerometer, continuous monitoring, real-world environments

## Abstract

Laboratory-based gait assessments are indicative of clinical outcomes (e.g., disease identification). Real-world gait may be more sensitive to clinical outcomes, as impairments may be exaggerated in complex environments. This study aims to investigate how different environments (e.g., lab, real world) impact gait. Different walking bout lengths in the real world will be considered proxy measures of context. Data collected in different dementia disease subtypes will be analysed as disease-specific gait impairments are reported between these groups. Thirty-two people with cognitive impairment due to Alzheimer’s disease (AD), 28 due to dementia with Lewy bodies (DLB) and 25 controls were recruited. Participants wore a tri-axial accelerometer for six 10 m walks in lab settings, and continuously for seven days in the real world. Fourteen gait characteristics across five domains were measured (i.e., pace, variability, rhythm, asymmetry, postural control). In the lab, the DLB group showed greater step length variability (*p* = 0.008) compared to AD. Both subtypes demonstrated significant gait impairments (*p* < 0.01) compared to controls. In the real world, only very short walking bouts (<10 s) demonstrated different gait impairments between subtypes. The context where walking occurs impacts signatures of gait impairment in dementia subtypes. To develop real-world gait assessment as a clinical tool, algorithms and metrics must accommodate for changes in context.

## 1. Introduction

Gait assessment may produce clinically meaningful outcomes, such as prediction and discrimination of neurological disorders from healthy controls [1,2,3,4,5], differentiation of dementia disease subtypes [3], monitoring progression of disease [6], reflecting cognitive impairments [7], identification of fall risk and freezing of gait [8,9,10,11], and assessment of treatment and rehabilitation efficacy [12,13]. While the majority of gait assessment occurs in clinical or lab-based environments, the advent of wearable technology has led to growing interest in continuously assessing gait in the real world in neurological populations such as those with dementia and Parkinson’s disease (PD). Wearable technology refers to mobile devices incorporating sensors which can be worn on the body or embedded into clothing, watches or bracelets. They can be worn continuously for extended periods of time, offering a novel and realistic perspective of gait [12]. There has been a significant growth in research examining real-world gait using wearable technology, with many studies employing a single tri-axial accelerometer on the body for unobtrusive data collection [8,14,15]. Accelerometers are a useful method of real-world data collection due to their inexpensive nature, ability to collect spatiotemporal and macro gait information (e.g., physical activity measures) and their timely set-up. 

It is hypothesised that real-world gait is more sensitive to disease-specific signatures of gait impairment compared to clinical and lab settings, due to the complexity and challenges of the environments [16]. While gait in the lab usually involves walking in a straight line at a consistent pace, real-world gait involves manoeuvring over different terrains, sharp and gradual turns and adaption of gait characteristics to differing environments. Therefore, measuring gait in a clinic or lab may only provide a narrow snapshot of performance capacity (i.e., what you can do in the best possible circumstances) as opposed to a continuous picture of true function (i.e., what you really do in a varying environment). Following this proposal, it has been suggested that real-world gait, as obtained by digital technology such as accelerometers, may provide more sensitive clinical endpoints for diagnostic and therapeutic purposes [15,17]. 

However, real-world gait analysis is still in its early stages and there is limited understanding surrounding “best practice” for data processing, analysis and interpretation [15]. One key area to gain a better understanding of is the impact of environment on gait and how to account for this when using gait as a clinical endpoint. This is integral to refining algorithms and applications of wearable technology for gait assessment in the real world. Initial comparisons of gait in the lab (measuring performance capacity) and in the real world (measuring true function) are required to form the base of this understanding. Additionally, considering the different contexts people move within may be a useful consideration for interpreting real-world data. Different walking bout lengths likely reflect different environmental contexts of walking. For example, if a person is walking from one room to the next, we may expect a short walking bout (<30 s), while walking outside in a community environment may be a period of sustained walking (>60 s; [15]). To choose the optimal environment and context for assessing gait for future clinical use, we must first understand how these circumstances impact signatures of gait impairment. 

This paper aims to investigate if gait assessments in both the lab and the real world can provide signatures of gait impairment that can discriminate between dementia disease subtypes and normal ageing, as assessed by wearable technology and if different walking bout lengths (serving as a proxy for real-world environmental contexts) can affect these signatures of gait impairment. It will report data from cognitively intact older adults and people with cognitive impairment due to Alzheimer’s disease (AD) and dementia with Lewy bodies (DLB), as these have previously been shown to have distinguishing signatures of gait impairment [3,18]. Therefore, we hypothesise that (1) DLB will demonstrate greater gait impairments compared to AD in all environments, (2) AD and DLB will demonstrate significant gait impairments compared to cognitively intact older adults when undertaking longer bout lengths in the real world, and (3) a greater number of gait characteristics will be impaired in dementia disease subtypes when in real-world environments compared to the lab. The intention of this exploratory cross-sectional study is to present gait data from both lab-based and real-world settings in dementia disease subtypes and controls, to allow for future generation of hypotheses, assessments and study protocols. Hypotheses will be explored through comparative analysis between the three groups. 

## 2. Materials and Methods

### 2.1. Study Participants

Participants included people with mild cognitive impairment (MCI) and probable dementia due to AD and DLB and similarly aged controls without any signs of cognitive impairment. Participants were recruited through a number of channels, including through Old Age Psychiatric, Geriatric Medicine or Neurology services after routine diagnostic assessments according to usual NHS practice, via the National Institute for Health Research Clinical Research Network portfolio, and by liaising with other ongoing clinical studies at Newcastle University to identify and approach potential suitable participants. 

Participants’ medical notes and assessments were reviewed by two independent clinicians who verified disease diagnosis, with a third clinician providing consensus diagnosis when disagreements arose. Formal diagnostic criteria for AD and DLB [19,20] and MCI [21,22] were used; MCI due to AD and Lewy body disease were identified as described in Donaghy [23] and King [24]. Inclusion and exclusion criteria for this study has previously been described in Mc Ardle [3]. This study was approved by the NHS Local Research Ethics Committee, Newcastle and North Tyneside. Reference: 16/NE/005, IRAS project ID: 192941.

### 2.2. Clinical and Cognitive Assessment

Age, sex, height, body mass and falls incidence in the last 12 months were recorded. The Cumulative illness rating scale—Geriatrics (CIRS-G) assessed co-morbidities, the National Adult Reading Test (NART) measured premorbid intelligence and the Bristol Activities of Daily Living Scale (BADLS) assessed impairments in activities of daily living. Movement Disorders Society Unified Parkinson’s Disease Rating Scale Part III (UPDRS-III) assessed severity of motor disease, and the Clinical Dementia Rating Scale (CDR) assessed dementia severity. Global cognition was measured using the standardised Mini Mental State Examination (sMMSE) and Addenbrookes Cognitive Examination III (ACE-III). 

### 2.3. Gait Assessment

**Figure 1:**
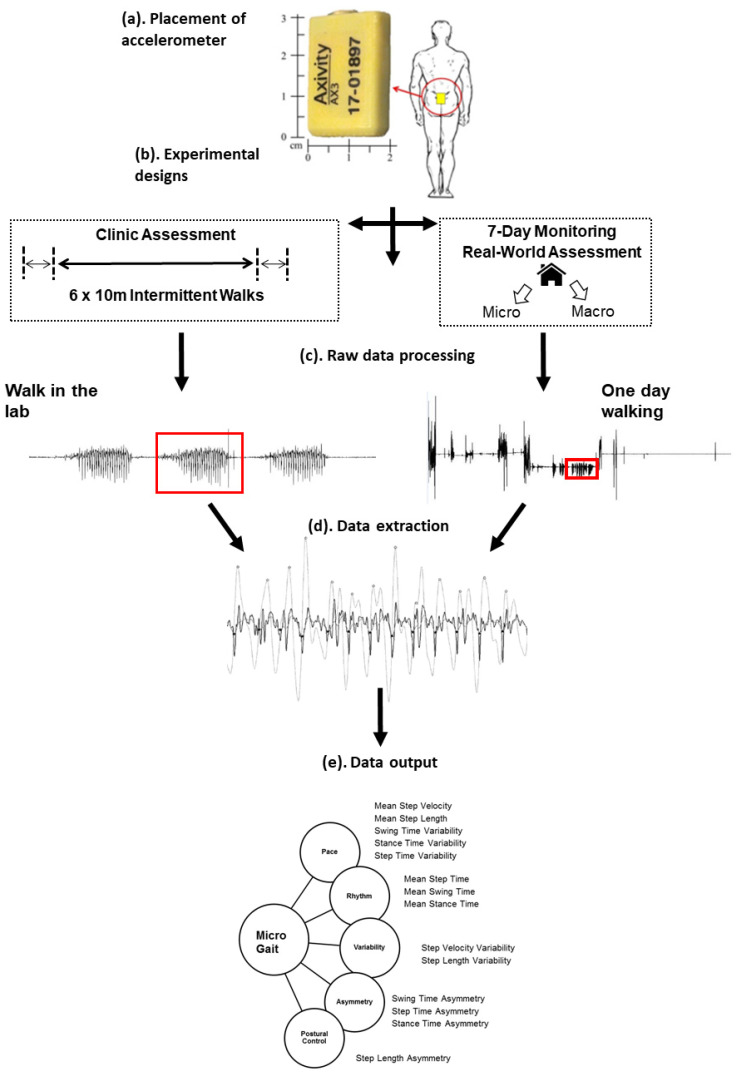
Illustration of gait protocol from initial body-worn monitor placement to data output, adapted from Mc Ardle [14]. (**a**) Example of body worn monitor placement for free-living data collection on L5 centrally located on the lower back; (**b**) gait protocols for lab and real-world assessment; (**c**) examples of lab and real-world accelerometer signals; (**d**) the raw vertical acceleration signal segmented into walking bouts; (**e**) conceptual model of gait representing domains and 14 gait characteristics. Figure adapted from Mc Ardle et al. (2018). The publication is available at IOS Press through DOI: 10.3233/JAD-171116.

For lab-based gait assessment, participants were asked to wear a body-worn monitor (Axivity AX3, York, UK; dimensions 23.0 × 32.5 × 7.6 mm; weight: 11 g; real-time accuracy/precision 20 parts per million) on their lower back. The monitor was attached to the skin above the fifth lumbar vertebra (L5) with double-sided tape and secured with Hypafix tape (see Figure 1). Participants were asked to carry out six ten-metre walks at their comfortable pace [3,18]. For real-world gait assessment, the body-worn monitor was placed on participants’ lower backs continuously for seven days [14].

### 2.4. Data Processing

For both lab-based and real-world gait, fourteen spatiotemporal gait characteristics were derived. Data from the body-worn monitors were downloaded to a computer and segmented by day. Analysis was carried out using a MATLAB^®^(2015A) programme. The full process from initial placement of the body-worn sensor through to data extraction and output is described in Figure 1. Accelerometer signals were transformed to a horizontal-vertical coordinate system and filtered with a 4th order Butterworth filter at 20 Hz in order to remove noise from the signal. Walking bouts were identified by applying selective thresholds on the magnitude of vector and the standard deviation of tri-axial acceleration signals (further detailed in Hickey [25]). For the real-world data processing, a minimum bout length of three consecutive steps was applied and a resting time threshold of 2.5 s—for example, if an individual stopped for longer than 2.5 s, their next three steps would be considered a new walking bout. This enhanced robustness and allowed us to remain consistent with previous published findings [2,14]. 

For each bout, the Gaussian continuous wavelet transform was applied to the vertical acceleration signal to allow identification of initial contact (heel strike) and final contact (toe-off) event of the gait cycle were identified, representing a step. From this, the mean time it takes to make a step can be calculated, subsequently allowing the calculation of mean stance and swing time [26]. Step length is calculated using the inverted pendulum model [27,28]. This approach uses the vertical motion of the trunk and monitor height (as a proxy for leg length) to estimate mean step length by assuming movement in the sagittal plane which approximately follows a sinusoidal motion during each single-leg stance phase. 

Step velocity was calculated from the ratio of step length to step time [27]. Variability of step, stance and swing time, step velocity and step length were determined by the standard deviations of all steps. Asymmetry was calculated using the absolute difference of consecutive steps (i.e., odd and even). These characteristics have been validated in the lab with measures obtained from the GaitRite Mat [26]; however, there was poor–moderate agreement with asymmetry and variability characteristics, which should be noted when interpreting results. 

For lab-based assessment, we considered each walking pass separately, while in the real-world data, we calculated gait characteristics for each discrete walking bout and then calculated the mean across all bouts [8,14,26].

### 2.5. Data Analysis

Data were assessed for normality by inspection of histograms and boxplots and the Shapiro–Wilk test. Levene’s test assessed homogeneity of variance. Chi-square tests determined differences between groups for gender and faller status (participants with and without falls during the previous year). One-way analysis of variance (ANOVA) and Kruskal Wallis tests examined differences between groups for all demographic, cognitive and clinical variables (determined by *p* < 0.05); where the differences lay was established using post-hoc analysis of independent *t*-tests and Mann Whitney U tests. In line with Aim One, we used one-way ANOVA and Kruskal Wallis tests to investigate if the three groups demonstrated between-group differences for each gait characteristic measured in the lab and the real world (encompassing all walking bouts). Post-hoc independent *t*-tests and Mann Whitney U tests were used to identify where the differences lay. Sensitivity analysis was conducted whereby results from both parametric and non-parametric tests were compared for non-normally distributed variables; where results did not change interpretation, results from one-way ANOVAs were reported. The value of statistical significance was set at (α = 0.01) in order to account for multiple comparisons; this was applied to both between-group analysis and subsequent post-hoc tests. A threshold of α = 0.01 was set a priori to aid interpretation of results and was a compromise between protecting against type I errors due to multiple corrections, and type II errors, given the exploratory nature of this study [29,30]. For full transparency and to allow the readers to assess the robustness of our interpretation, we have provided unadjusted α values up to 3 decimal points in our results section. In line with Aim Two, we used one-way ANOVA and Kruskal Wallis tests, where appropriate, to explore between-group differences for each gait characteristic measured in different walking bout lengths. This encompassed four discrete bout lengths: in only very short real-world walking bouts (<10 s), in only real-world short walking bouts (<30 s), in only medium real-world walking bouts (30–60 s), and in only long real-world walking bouts (>60 s). Similarly, post-hoc independent *t*-tests and Mann Whitney U tests identified where the differences lay, and statistical significance was set at (α = 0.01). 

We also undertook secondary analysis using Pearson’s correlations to identify associations between characteristics for lab-based and real-world gait to aid interpretation of data. 

## 3. Results

### 3.1. Participants

In total, 125 participants were recruited to the study; only AD, DLB and control participants with both lab-based and real-world gait assessment were included in this analysis (n = 85). This left 32 with cognitive impairment due to AD, 28 due to DLB, and 25 controls. Groups were composed of predominately mild dementia cases (see Table 1 for all clinical and demographic information). All participants had capacity to consent to participation in the study in accordance with the Mental Capacity Act [31], and provided written informed consent.

### 3.2. Proportion of Complete Datasets

For the lab-based gait assessment, six participant datasets (6%) were lost due to user error, such as device not syncing (n = 3), wrong trial time recorded (n = 2), and accelerometer not turned on (n = 1). For the real-world gait assessment, four participant datasets (4%) were lost due to problems with data upload (n = 2), refusal to wear the monitor (n = 1) and the monitor being lost in the post (n = 1). Therefore, we included 89% of the original datasets collected in participant’s with AD, DLB and controls. 

From the included datasets, five participants (6%) had less than seven days data collected due to hospitalisation (n = 1), discomfort (n = 1) and quality checks (n = 3). All participants had over three days of data collected; 3–7 days of data collection is the current standard of free-living gait analysis [15]. 

### 3.3. Does Lab-Based and Real-World Gait Assessment Produce Similar Signatures of Gait Impairment?

#### 3.3.1. Lab-Based Gait Assessment

In the lab, people with DLB demonstrated significantly greater step length variability (*p* = 0.008) compared to people with AD (see Table 2). Both dementia disease groups were more variable for step, stance and swing time (*p* < 0.01), spent longer in swing time (<0.01) and had greater swing time asymmetry (*p* < 0.01) in comparison to controls. Additionally, people with DLB walked slower (*p* = 0.010), with greater step velocity variability (*p* < 0.001) and longer step time (*p* = 0.001) compared to controls. 

#### 3.3.2. Real-World Gait

When considering all bouts over three steps in the real world, there were no significant differences between AD and DLB or between AD and controls at the threshold of *p* ≤ 0.01 (see Table 2). People with DLB walked slower (*p* < 0.001) with shorter steps (*p* < 0.001), and greater variability for step (*p* = 0.002), swing (*p* = 0.002) and stance time (*p* = 0.001) compared to controls.

#### 3.3.3. Associations between Lab-Based and Real-World Gait

There were moderate–positive correlations between performance in the lab and the real world for pace (step velocity (r = 0.530, *p* < 0.001), step length (r = 0.589, *p* < 0.001)), rhythm (step time (r = 0.523, *p* < 0.001), swing time (r = 0.522, *p* < 0.001), stance time (r = 0.552, *p* < 0.001)) and asymmetry (step time asymmetry (r = 0.451, *p* < 0.001), swing time asymmetry (r = 0.476, *p* < 0.001) and stance time asymmetry (r = 0.499, *p* < 0.001)). 

However, there were no correlations (*p* > 0.05) between the lab and the real world for any characteristics pertaining to variability or postural control (step length asymmetry). When examining trends, all groups appear to walk faster with longer steps, greater variability and asymmetry and longer timing of gait in the real world compared to in the lab. 

### 3.4. Do Discrete Bout Lengths Impact Discriminative Signatures of Gait Impairment in the Real World?

**<10 s walking bouts**

**Figure 2:**
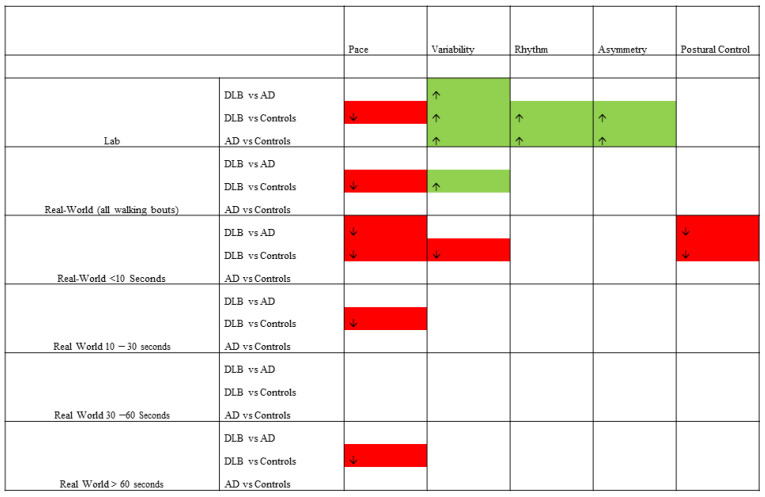
Gait impairments between groups across all conditions. Green = characteristic is significantly greater in the first group than the second. Red = characteristic is significantly less in the first group than the second.

When considering very short walking bouts, people with DLB demonstrated significantly shorter steps (*p* = 0.007) and less step length asymmetry (*p* = 0.002) compared to the AD group (see Table 3). They also had shorter steps (*p* < 0.001), less step length variability (*p* = 0.002) and less step length asymmetry (*p* = 0.003) compared to controls. There were no differences between the AD group and controls. All results are illustrated in Figure 2. 

**10–30 s walking bouts**

In short walking bouts, people with DLB had significantly shorter steps (*p* <0.001) than controls (see Table 3). However, there were no other group differences found (*p* ≤ 0.01).

**30–60 s walking bouts**

There were no differences in gait impairment between groups during medium walking bouts (*p* ≤ 0.01; see Table 3). 

**>60 s walking bouts**

In sustained walking bouts, people with DLB walked slower (*p* = 0.003) compared to controls; there were no other differences between groups (*p* > 0.01; see Table 3).

Associations between Lab-Based and Real-World Gait Characteristics within Discrete Bout Lengths.

**<10 s walking bouts**

There were weak–moderate correlations between gait in the lab and gait in the real world for very short walking bouts in the following characteristics: pace (step length (r = 0.360; *p* < 0.001)), rhythm (stance time (r = 0.224, *p* = 0.041)) and asymmetry (swing time asymmetry (r = 0.420; *p* < 0.001) and stance time asymmetry (r = 0.410, *p* < 0.001)). 

**10–30 s walking bouts**

There were weak–moderate correlations between gait in the lab and gait in the real world for short walking bouts in the following characteristics: pace (step velocity (r = 0.289; *p* = 0.008), step length (r = 0.496; *p* < 0.001)), rhythm (step time (r = 0.319; *p* = 0.003), swing time (r = 0.276; *p* = 0.011), stance time (r = 0.411, *p* < 0.001)) and asymmetry (step time asymmetry (r = 0.259, *p* = 0.017), swing time asymmetry (r = 0.452; *p* < 0.001) and stance time asymmetry (r = 0.409; *p* < 0.001)).

**30–60 s walking bouts**

There were weak–moderate correlations between gait in the lab and gait in the real world for medium walking bouts in the following characteristics: pace (step velocity (r = 0.348; *p* < 0.001), step length (r = 0.569; *p* < 0.001)), rhythm (step time (r = 0.356; *p* < 0.001), swing time (r = 0.341; *p* = 0.002), stance time (r = 0.424, *p* < 0.001)) and asymmetry (step time asymmetry (r = 0.397, *p* < 0.001), swing time asymmetry (r = 0.486; *p* < 0.001) and stance time asymmetry (r = 0.501; *p* < 0.001)).

**>60 s walking bouts**

There were weak–moderate correlations between gait in the lab and gait in the real world for sustained walking bouts in the following characteristics: pace (step velocity (r = 0.548; *p* < 0.001), step length (r = 0.641; *p* < 0.001)), rhythm (step time (r = 0.535; *p* < 0.001), swing time (r = 0.542; *p* = 0.002), stance time (r = 0.539, *p* < 0.001)) and asymmetry (step time asymmetry (r = 0.286, *p* = 0.008), swing time asymmetry (r = 0.437; *p* < 0.001) and stance time asymmetry (r = 0.377; *p* < 0.001)).

## 4. Discussion

The main aim of this paper was to investigate if assessing gait with wearable technology in the lab and the real world provides similar signatures of gait impairment that discriminate between dementia disease groups and normal ageing, and to consider the impact of different walking bout lengths, as proxy measures of different contexts, on patterns of gait impairment. Data from people with AD, DLB and cognitively intact older adults were reported, as disease-specific signatures of gait impairment has previously been demonstrated [3]. Contrary to speculations in the literature [15], results from this study indicate that gait impairments measured in controlled lab-based conditions may be better at distinguishing between AD and DLB, and between AD and controls, regardless of walking bout length. Therefore, gait in the lab and the real world depict different patterns of gait impairment, regardless of walking bout length. 

### 4.1. The Impact of Environment on Gait

As expected and predicted by Hypotheses 1 and 2, people with DLB had significantly greater gait impairment than the AD group and both disease groups had impaired patterns of gait compared to controls. However, in contrast to Hypothesis 3, these findings were most evident in controlled laboratory settings; there were no significant differences between AD and controls during real-world gait, and only very short walking bouts showed differences between DLB and AD. This brings back the question of performance capacity versus true function, and how these differing strategies and environments may affect gait. In laboratory settings, participants are asked to attend to the gait task and may improve their performance compared to their real-world gait. However, this may depend on cognitive capacity as gait requires significant interaction with complex cognitive processes such as executive function and attention [7,32], and people with cognitive impairment may not be able to improve their gait to the same degree as similarly aged cognitively intact individuals. Control participants may have greater capacity to cognitively engage with gait compared to AD participants, and therefore their “best” gait performance underlies the significant differences in gait performance between these groups. In real-world environments, controls are demonstrating their “normal” gait without obtrusive observation; thus, their daily function may more closely align to that of AD. Future research should consider the diverse applications of assessing performance capacity and true function, as discrete questions may require different methods, such as assessing impairments in performance capacity to detect cognitive impairment. 

Surprisingly, this study demonstrates that monitoring real-world gait could only identify impairments in people with DLB. Exploring gait in different walking bout lengths did appear to reveal different patterns of gait impairment; longer walking bouts demonstrated less differences in gait between groups. Perhaps the added environmental complexity of the real world actually makes it more difficult to discern unique signatures of gait between different pathologies. Real-world settings experienced between our participants are not necessarily comparable. For example, it is suggested that longer walking bouts would more likely take place in environments outside of the home [2]. For one participant, this may require navigation around unkempt pavements or walking up steep slopes; for another, it may be walking on a straight flat surface. Both environments are likely to impact gait differently [33], and potentially explain why there were limited differences between groups in the real world. When all participants carry out the same walking task in the same environment such as a lab, the different patterns of gait impairment may be more likely to be disease-specific or cognitively driven. When we monitor gait in the real world across a range of varying complex environments, the differing environments themselves may significantly confound our results. It is important to acknowledge such limitations when choosing which method of gait analysis to use, as lab-based and real-world gait may be better suited to different purposes. Lab-based gait may be best applied when examining between-group differences, such as differentiating disease subtypes [18] while real-world gait may be useful to monitor within-person changes such as disease progression and responses to therapeutics or interventions [15].

Speculatively, such confounders may explain why there were no significant associations between measures of gait variability in the lab and the real world. Gait variability may be particularly sensitive to environmental properties or dual-tasking inherent to everyday life and change accordingly, and in turn, may have muted differences shown in the lab between AD and controls, and AD and DLB. As gait variability is a key difference between dementia disease subtypes, and is reflective of “higher-order” cognitive processes, such as attention and executive function [3,7], it may be important to optimally assess this variable. This is not to say that real-world gait should not be assessed, as it has potential to decrease numbers of outpatient visits and reduce healthcare costs, provide objective evidence to tailor and assess therapies and interventions, and improve how we deliver high-quality care and precision medicine [34]. Unexpected results such as these simply serve to remind us that there are still limitations to real-world gait assessment that need to be prioritised and addressed. 

### 4.2. Challenges and Future Directions for the Application of Wearable Technology to Assess Real-World Gait

One of the key challenges to advancing real-world gait analysis is translating wearable algorithms designed for and validated in controlled indoor experiments for use in real-world settings [4,26]. These algorithms place a number of assumptions on experimental design, such as exact position of sensors and type of walking terrain. They do not account for changes in environment or gait speed. Some algorithms have been developed by simulating real-world environments, but their performance has yet to assessed for longer periods of time [35]. The unobtrusive nature of wearable devices also means that in these early stages of real-world gait assessment, we do not fully understand the impact of context on discrete gait impairments. For example, in this study, people with DLB were less variable and asymmetric compared to AD and controls in very short walking bouts—an unexpected finding which is not easily interpretable without understanding how they interact with environments experienced in very short walking bouts. The strongest correlations between lab and real-world gait metrics were also found in the longer walking bouts, suggesting the algorithm performs better in continuous walking—which only accounts for a small proportion of total data collected. As such, we need to gain a better understanding about how environmental context impacts gait, beyond considering length of walking bouts [36]. This could be done through initial validation with wearable cameras, and less obtrusively with companion applications such as mobile GPS or assessment of the walkability of the participants’ usual environment [37]. 

Additionally, there is yet to be a consensus on the best metrics to describe gait in the real world. There are a wealth of alternative features of gait beyond those described in this paper, such as frequency-based [12,38] and ambulatory activity metrics [39] alongside emerging characteristics from gyroscopes applied to the real world [40], making it difficult to pinpoint the most relevant characteristics within the literature. We recommend that collaborative approaches should be adopted to move beyond small, isolated pilot studies with a variety of metrics in order to accumulate and condense data, select target characteristics and encourage “best-practice” standards for application and analysis of wearables in clinical practice [34]. These collaborations should also engage with clinicians, patients and regulatory bodies to ensure validated approaches are clinically relevant, easy to use, cost-effective and contribute to clinical decisions beyond what can already be decided from careful neurological examination and clinical phenotyping [41]. Target characteristics should account for or be independent of environment and context, and may need to characterise fluctuations in gait throughout the day as this may provide a more realistic picture of functional abilities and disease progression [42]. As our knowledge advances, we may need to consider real-world gait separately from lab-based gait, and understand which method is most appropriate to answer discrete clinical questions. 

### 4.3. Strengths and Limitations of the Current Study

A key strength of the current study is the acquisition of a well-characterised dementia disease sample, whose disease subtype was ascertained via clinical consensus. This allows greater confidence in our findings, although it should be noted that dementia diagnosis can only be confirmed post-mortem. While this is the first study to report real-world gait in AD and DLB, interpretation of results is limited by our small sample size. Due to the exploratory nature of this study, we did not use stringent methods, such as Bonferroni corrections, to account for multiple comparisons; as such, our results should be used to guide future hypotheses in larger cohort studies to validate our findings. Additionally, the algorithm employed in this study has previously been shown to exaggerate characteristics of variability and asymmetry when compared to the same metrics derived from an instrumented walkway [18,26]. This may bring in the question of validity, and it could be argued that using different or multiple sensor placements, such as one on the lower back and two on the feet, or different devices, such as inertial measurement units or Kinect, may provide more confidence in gait characteristics derived [43]. We chose to use a single accelerometer placed on the lower back as this is less burdensome to wear for continuous monitoring of gait, and the algorithm has been validated to accurately detect walking behaviours in the real world [25]. However, the potential limitations that come with this approach highlight the need for standardised assessment and reporting protocols in this field. As noted, we are unaware of the environments that participants moved within for the duration of the study; future work could explore this by asking participants to keep a diary of their movements or by integrating GPS devices into the study protocol. Different pathologies may require different protocols and outcomes, and this should also be considered in future research. Finally, our study results may have encountered social desirability bias, as participants may have changed their walking behaviours due to wearing the device. As participants wore the device continuously for seven days, habituation may have reduced this bias, but without a follow-up questionnaire, we cannot confirm this. Future research should examine this prospect in gait performance. 

## 5. Conclusions

This study demonstrated that gait impairments assessed by wearable technology are dependent on the environment in which walking takes place. Using wearable technology in the lab may be useful as a clinical tool for differential diagnosis of dementia subtypes. However, more research surrounding the role of environment and context is required to translate these findings to real-world gait. Collaborative approaches are needed to address these issues and studies such as this one may be more fruitful as strengthened algorithms are applied retrospectively. 

## Figures and Tables

**Table 1:** Demographic, clinical and cognitive information for all groups.

				Differences between All Groups	
	Controls	AD	DLB	F/X^2^	P
N	25	32	28		
Age	74 ± 9	77 ± 6	76 ± 6	0.9	0.405
Gender (m/f)	11/14 ^D^	15/17 ^D^	22/6 ^A,C^	8.3 (138) *	**0.015**
Height (m)	1.67 ± 0.10	1.67 ± 0.11	1.70 ± 0.10	1.5 †	0.464
Weight (kg)	75.6 ± 16.4	72.3 ± 12.2	78.0 ± 12.8	2.4 †	0.300
BMI	26.83 ± 4.33	26.03 ± 4.62	27.24 ± 4.82	0.1	0.833
Faller (Yes/No)	5/19 ^A,D^	15/17 ^C^	17/11 ^C^	8.5 *	**0.014**
NART	122 ± 3 ^A,D^	117 ± 6 ^C^	115 ± 6 ^C^	23.3 †	**<0.001**
sMMSE (0–30)	29 ± 1 ^A,D^	23 ± 4 ^C^	24 ± 4 ^C^	44.5 †	**<0.001**
ACE-III (0–100)	96 ± 3 ^A,D^	72 ± 13 ^C^	73 ± 18 ^C^	50.0 †	**<0.001**
CDR (0–3)	0 ± 0 ^A,D^	0.8 ± 0.3 ^C^	0.8 ± 0.3 ^C^	70.0	**<0.001**
UPDRS (0–108)	2 ± 3 ^A,D^	7 ± 6 ^C,D^	23 ± 15 ^C,A^	43.2 †	**<0.001**
CIRS-G	5 ± 3 ^A,D^	8 ± 4 ^C^	10 ± 4 ^C^	21.8 †	**<0.001**
GDS	1 ± 1 ^A,D^	4 ± 3 ^C^	5 ± 3 ^C^	29.2 †	**<0.001**
ABC	93 ± 10 ^A,D^	80 ± 18 ^C^	81 ± 17 ^C^	12.9 †	**0.002**
BADLS	0 ± 0 ^A,^*^,D^	9 ± 7 ^C^	13 ± 6 ^C^	43.8 †	**<0.001**

BMI = Body Mass Index, NART = National Adult Reading Test, sMMSE = Standardised Mini Mental State Examination, CDR = Clinical Dementia Rating Scale, UPDRS-III = Unified Parkinson’s Disease Rating Scale III, CIRS-G = Cumulative Illness Rating Scale—Geriatrics, GDS = Geriatric Depression Scale, ABC = Activity Balance Confidence Scale, BADLS = Bristol Activities of Daily Living Scale. P value in the table represents difference between groups derived from Kruskal Wallis tests, ANOVA or chi-square tests; * marks results determined from chi-square tests, † indicates where results were determined from Kruskal Wallis tests. Degrees of freedom relating to ANOVA and chi-square results = 2. Annotations within table represents differences between groups from *t*-tests, Mann Whitney U tests, or chi-square tests and are interpreted as follows: C = different to controls, A = different to AD, D = different to DLB.

**Table 2:** Differences in gait impairment between dementia disease groups and controls in both lab and real-world settings.

Lab-Based Gait	Controls	AD	DLB	F	*p*	Sig	Real-World Gait	Controls	AD	DLB	F	*p*	Sig
**N**	**25**	**32**	**28**					**25**	**32**	**28**			
**Mean step count**	109	115	11					99,224	78,794	72,482			
**Pace**							**Pace**						
Step Velocity (m/s)	1.02 ± 143	0.90 ± 0.17	0.92 ± 0.13	9.05	0.014		Step Velocity (m/s)	1.08 ± 0.08	1.02 ± 0.11	0.98 ± 0.10	7.2	**0.001**	**b**
Step Length (m)	0.55 ± 0.08	0.52 ± 0.08	0.54 ± 0.065	1.7	0.193		Step Length (m)	0.61 ± 0.04	0.58 ± 0.05	0.56 ± 0.05	7.2	**0.001**	**b**
Step Time SD (s)	0.026 ± 0.015	0.043 ± 0.025	0.062 ± 0.045	9.1	**<0.001**	**a,b**	Step Time SD (s)	0.166 ± 0.014	0.175 ± 0.018	0.177 ± 0.016	4.8	**0.010**	**b**
Swing Time SD (s)	0.021 ± 0.009	0.042 ± 0.024	0.053 ± 0.030	12.8	**<0.001**	**a,b**	Swing Time SD (s)	0.140 ± 0.012	0.146 ± 0.015	0.152 ± 0.014	5.0	**0.009**	**b**
Stance Time SD (s)	0.027 ± 0.013	0.048 ± 0.027	0.065 ± 0.042	10.7	**<0.001**	**a,b**	Stance Time SD (s)	0.177 ± 0.016	0.187 ± 0.019	0.193 ± 0.018	5.2	**0.007**	**b**
**Variability**							**Variability**						
Step Length SD (m)	0.043 ± 0.019	0.060 ± 0.026	0.082 ± 0.034	13.4	**<0.001**	**a,b,c**	Step Length SD (m)	0.149 ± 0.017	0.147 ± 0.013	0.153 ± 0.008	1.5	0.226	
Step Vel SD (m/s)	0.084 ± 0.032	0.107 ± 0.045	0.142 ± 0.057	10.6	**<0.001**	**b**	Step Vel SD (m/s)	0.359 ± 0.033	0.358 ± 0.033	0.369 ± 0.037	0.8	0.445	
**Rhythm**							**Rhythm**						
Step Time (s)	0.543 ± 0.048	0.576 ± 0.056	0.594 ± 0.060	5.8	**0.004**	**b**	Step Time (s)	0.594 ± 0.030	0.604 ± 0.024	0.603 ± 0.030	1.1	0.333	
Swing Time (s)	0.387 ± 0.041	0.423 ± 0.053	0.441 ± 0.064	7.0	**0.002**	**a,b**	Swing Time (s)	0.445 ± 0.029	0.458 ± 0.026	0.457 ± 0.028	0.9	0.396	
Stance Time (s)	0.699 ± 0.059	0.727 ± 0.067	0.744 ± 0.066	3.3	0.042		Stance Time (s)	0.743 ± 0.034	0.754 ± 0.025	0.752 ± 0.035	1.9	0.150	
**Asymmetry**							**Asymmetry**						
Step Time Asy (s)	0.024 ± 0.016	0.034 ± 0.023	0.034 ± 0.019	2.3	0.112		Step Time Asy (s)	0.093 ± 0.008	0.099 ± 0.013	0.095 ± 0.008	2.7	0.073	
Swing Time Asy (s)	0.021 ± 0.012	0.039 ± 0.029	0.034 ± 0.020	5.2	**0.007**	**a,b**	Swing Time Asy (s)	0.086 ± 0.008	0.090 ± 0.011	0.089 ± 0.009	1.8	0.179	
Stance Time Asy (s)	0.021 ± 0.012	0.039 ± 0.027	0.034 ± 0.019	4.7	0.011		Stance Time Asy (s)	0.095 ± 0.008	0.100 ± 0.013	0.096 ± 0.010	2.4	0.096	
**Postural Control**							**Postural Control**						
Step Length Asy (m)	0.067 ± 0.039	0.104 ± 0.070	0.088 ± 0.061	2.7	0.076		Step Length Asy (m)	0.086 ± 0.007	0.089 ± 0.012	0.082 ± 0.010	3.4	0.040	

*p* value in table represents differences between all groups as reported from ANOVA. Statistical significance set at α = 0.01. Sig annotations within table represents differences between groups from *t*-tests and are interpreted as follows: a = differences between AD and controls, b = difference between DLB and controls, c = differences between AD and DLB. Other abbreviations include: SD = within-person standard deviation (i.e., gait variability), asy = asymmetry, m = metre, s = second, ms = millisecond.

**Table 3:** Differences in gait impairment between groups in discrete walking bout lengths in the real world.

	<10 s Bouts				10–30 s Bouts			
	Controls	AD	DLB	(*p*)	Controls	AD	DLB	(*p*)
Bouts per day	359 ± 102	356 ± 151	339 ± 126	0.723	199 ± 59	195 ± 70	186 ± 74	0.697
**Steps per day**	1775 ± 546	1763 ± 735	1784 ± 700	0.992	3694 ± 1093	3660 ± 1314	3583 ± 1424	0.950
**Pace**								
Step Velocity (m/s)	0.91 ± 0.06	0.89 ± 0.09	0.86 ± 0.08	0.062	1.01 ± 0.06	0.98 ± 0.09	0.96 ± 0.09	0.019 †
Step Length (m)	0.53 ± 0.02	0.52 ± 0.03	0.49 ± 0.03	**≤** **0.001 †**	0.58 ± 0.03	0.56 ± 0.04	0.54 ± 0.04	**0.001 †**
Swing SD (s)	0.170 ± 0.013	0.169 ± 0.012	0.170 ± 0.012	0.951	0.156 ± 0.012	0.155 ± 0.015	0.160 ± 0.015	0.336
Step Time SD (s)	0.206 ± 0.013	0.207 ± 0.01	0.205 ± 0.014	0.564 †	0.183 ± 0.014	0.184 ± 0.016	0.189 ± 0.018	0.702 †
Stance SD (s)	0.220 ± 0.013	0.220 ± 0.014	0.220 ± 0.014	0.881 †	0.195 ± 0.014	0.196 ± 0.016	0.202 ± 0.020	0.263
**Variability (SD)**								
Step Velocity SD (m/s)	0.384 ± 0.027	0.376 ± 0.044	0.370 ± 0.042	0.276 †	0.380 ± 0.030	0.372 ± 0.039	0.379 ± 0.042	0.588
Step Length SD (m)	0.163 ± 0.007	0.158 ± 0.009	0.156 ± 0.009	**0.009 †**	0.153 ± 0.009	0.151 ± 0.009	0.153 ± 0.008	0.548 †
**Rhythm**								
Step Time (ms)	0.616 ± 0.022	0.614 ± 0.029	0.602 ± 0.025	0.107	0.618 ± 0.027	0.618 ± 0.030	0.610 ± 0.030	0.476
Swing (ms)	0.472 ± 0.023	0.471 ± 0.029	0.461 ± 0.023	0.163 †	0.473 ± 0.028	0.476 ± 0.32	0.468 ± 0.029	0.560
Stance (ms)	0.764 ± 0.024	0.763 ± 0.031	0.750 ± 0.030	0.131 †	0.767 ± 0.028	0.765 ± 0.030	0.758 ± 0.035	0.516
**Asymmetry**								
Step Time Asy (ms)	0.162 ± 0.012	0.170 ± 0.021	0.160 ± 0.013	0.086 †	0.072 ± 0.007	0.075 ± 0.012	0.072 ± 0.009	0.573 †
Swing Asy (ms)	0.123 ± 0.010	0.127 ± 0.017	0.122 ± 0.012	0.765 †	0.067 ± 0.007	0.070 ± 0.011	0.067 ± 0.009	0.526 †
Stance Asy (ms)	0.165 ± 0.011	0.171 ± 0.019	0.162 ± 0.014	0.132 †	0.073 ± 0.007	0.076 ± 0.012	0.073 ± 0.009	0.565 †
**Postural Control**								
Step Length Asy (m)	0.121 ± 0.010	0.121 ± 0.015	0.110 ± 0.014	**0.002 †**	0.081 ± 0.008	0.080 ± 0.013	0.073 ± 0.012	0.014 †
	**30–60 s bouts**				**>60 s bouts**			
	**Controls**	**AD**	**DLB**	**(*p*)**	**Controls**	**AD**	**DLB**	**(*p*)**
Bouts per day	42 ± 15	38 ± 16	36 ± 19	0.311	26 ± 12	18 ± 8	16 ± 11	0.011
Steps per day	2105 ± 715	1846 ± 788	1783 ± 956	0.334	6601 ± 3621	4228 ± 2956	3205 ± 2786	**0.001**
**Pace**								
Step Velocity (m/s)	1.05 ± 0.06	1.01 ± 0.08	1.00 ± 0.11	0.112	1.15 ± 0.11	1.08 ± 0.13	1.05 ± 0.13	**0.010**
Step Length (m)	0.60 ± 0.03	0.58 ± 0.04	0.57 ± 0.04	0.019 †	0.63 ± 0.06	0.60 ± 0.06	0.59 ± 0.06	0.044
Swing SD (s)	0.149 ± 0.010	0.151 ± 0.015	0.155 ± 0.018	0.283	0.114 ± 0.019	0.125 ± 0.025	0.129 ± 0.025	0.069
Step Time SD (s)	0.175 ± 0.012	0.179 ± 0.019	0.183 ± 0.022	0.263	0.137 ± 0.024	0.149 ± 0.033	0.153 ± 0.032	0.119
Stance SD (s)	0.186 ± 0.013	0.190 ± 0.021	0.195 ± 0.023	0.225	0.147 ± 0.027	0.161 ± 0.036	0.153 ± 0.032	0.110
**Variability (SD)**								
Step Velocity SD (m/s)	0.366 ± 0.030	0.364 ± 0.037	0.372 ± 0.042	0.706	0.316 ± 0.054	0.320 ± 0.063	0.329 ± 0.058	0.719
Step Length SD (m)	0.150 ± 0.011	0.148 ± 0.012	0.151 ± 0.010	0.702 †	0.132 ± 0.028	0.133 ± 0.025	0.138 ± 0.020	0.534 †
**Rhythm**								
Step Time (ms)	0.614 ± 0.027	0.615 ± 0.025	0.612 ± 0.036	0.934	0.570 ± 0.042	0.588 ± 0.031	0.594 ± 0.39	0.161 †
Swing (ms)	0.466 ± 0.026	0.470 ± 0.025	0.467 ± 0.034	0.876	0.419 ± 0.035	0.437 ± 0.027	0.443 ± 0.036	0.023
Stance (ms)	0.763 ± 0.029	0.764 ± 0.026	0.761 ± 0.042	0.920	0.719 ± 0.050	0.737 ± 0.036	0.746 ± 0.044	0.194 †
**Asymmetry**								
Step Time Asy (ms)	0.040 ± 0.005	0.042 ± 0.007	0.043 ± 0.006	0.327 †	0.023 ± 0.006	0.027 ± 0.006	0.027 ± 0.007	0.040 †
Swing Asy (ms)	0.036 ± 0.005	0.038 ± 0.006	0.038 ± 0.006	0.206 †	0.021 ± 0.005	0.025 ± 0.006	0.025 ± 0.006	0.022 †
Stance Asy (ms)	0.040 ± 0.005	0.042 ± 0.007	0.042 ± 0.007	0.410 †	0.023 ± 0.005	0.027 ± 0.006	0.026 ± 0.007	0.017 †
**Postural Control**								
Step Length Asy (m)	0.048 ± 0.006	0.50 ± 0.010	0.047 ± 0.009	0.308 †	0.026 ± 0.007	0.031 ± 0.015	0.032 ± 0.009	0.040

*p* value in table represents differences between all groups as reported from ANOVA/Kruskal Wallis tests. † indicates where results were determined from Kruskal Wallis tests. Statistical significance set at α = 0.01. Other abbreviations include: SD =within-person standard deviation (i.e., gait variability), asy = asymmetry, m = metre, s = second, ms = millisecond.

## Data Availability

The data presented in this study are available on request of the corresponding author. The data are not publicly available due to ongoing analysis and publication.
